# Cardiovascular Disease as a Risk Factor for Depression in Korean Older Persons

**DOI:** 10.1093/geroni/igaf122.3891

**Published:** 2025-12-31

**Authors:** Heashoon Lee

**Affiliations:** Hannam University, Daejeon, Republic of Korea

## Abstract

As populations live longer, supporting emotional well-being alongside physical health is essential to healthy aging. This study explores how specific cardiovascular diseases (CVDs) are associated with depressive symptoms among Korean older persons. While earlier studies often examined depression as a risk factor for CVD, this research reverses that approach—investigating CVD as a predictor of late-life depression using newly available national data. Data were drawn from 1,648 Korean people aged 65 years and older, using the 2023 Korea National Health and Nutrition Examination Survey (KNHANES), which became publicly available after the general GSA abstract deadline. Analyses used a complex sampling design and included t-tests, Rao–Scott chi-square tests, and multiple logistic regression adjusted for relevant covariates. Results showed that older persons diagnosed with angina pectoris were 3.62 times more likely to report depressive symptoms, those with dyslipidemia were 1.91 times more likely, and those with stroke were 2.32 times more likely, compared to those without these conditions. In contrast, hypertension, systolic/diastolic blood pressure, and myocardial infarction were not significantly associated with depression. Additionally, women were 3.03 times more likely than men to experience depressive symptoms. These findings underscore the value of integrating cardiovascular and mental health services in age-inclusive care models. Tailored interventions for those living with angina pectoris, dyslipidemia, or stroke may enhance both emotional well-being and quality of life for older people in diverse aging societies.

